# Reviews of Books

**Published:** 1922-03

**Authors:** 


### REVIEWS OF BOOKS.

**PHYSIC AND FICTION.** By S. SQUIRE SPRIGGE. (Hodder & Stoughton; 12s. 6d. net.)

Perhaps Sir Squire Sprigge has not chosen the best possible title for his collection of essays on medical subjects, which are by no means confined to a rapid and able survey of the various doctors described by Dickens, Thackeray and others. He is quite as much concerned with fact as fiction and treats in the fashion of an able publicist some of the burning questions in medical jurisprudence and sociology of the present day. For instance, in discussing the cases of the last four medical criminals who have suffered the capital penalty—Palmer, Pritchard, Lamson and Neill-Cream, he is at pains to show that no foundation exists for the vague popular superstition that we laymen lie innocently at the mercy of the special knowledge of poisons possessed by the profession. Analysing each case, he concludes that public justice is better equipped with expert knowledge than any practitioner who is ever likely to be tempted to attack instead of defending human life. Again, he opens and, if I may say so, convincingly closes, an inquiry now prevalent among eugenic circles, if not in a wider public, as to the advisability or possibility of enforcing by legislation a health certificate from all candidates for marriage. Distinguishing between new and settled countries like our own he decides that for the latter the social, not to say individual, inconveniences and evasions attending compulsion would import an evil far outweighing any possible benefit from such an attempt to regularise the mating of human identities. Even in face of so obvious a danger as blood-poisoning by syphilis, many and often the most glaring cases would escape any penalty or restriction, while the necessary inquisition would amount to an unmerited burden on the innocent. As to greater foes to health in diseases like cancer and tuberculosis, he concludes that existing medical knowledge on the method and possibility of transmission is far from sufficient to afford or justify decisions both authoritative and inexpugnable, such as legal regulation would require. On more purely professional topics, such as a comparison between a doctor's profession and other careers in the balance between rewards and failures, he has assembled much useful statistics, notably some investigations originally started by Sir James Paget in the life history of 1,000 students at Bart's and continued by others from the records of St. George's and St. Thomas's Hospitals. These figures confirm the natural belief that the prospects of a young man taking up medicine as a livelihood are well on a par with most of those offered elsewhere. In those chapters of the book which deal with fiction perhaps his best illustrations are taken from his analysis of a case of heart-disease in Guy de Maupassant's "Une Vie," and his elaborate parallel between the character of Dr. Goodenough, drawn by Thackeray, and a real personage of the time, Dr. John Elliotson, the famous physician at University College Hospital. The latter has sometimes been suggested as having been the original of the shady career of the former, and from this unfair comparison the author is at pains to vindicate Elliotson's memory.—G. B. D.

**THE PSYCHO-ANALYTIC STUDY OF THE FAMILY.** By J. C. FLÜGEL. (Allen & Unwin; 10s. 6d. net.)

This book, which is the third volume of the series known as the International Psycho-Analytical Library, is a careful endeavour to apply the methods of psycho-analysis, hitherto mainly devoted to the study of the unconscious motives in individuals, to the study of family life. The relations of parents to children and of brothers to sisters are examined, because a child's attitude toward these largely determines his attitude in adult life to his fellow-men. The family is society in little; all human relations spring from and are analogous to family relations. It is, therefore, a storm centre of emotions and desires, many of which, from the nature of the case, do not emerge into the individual's consciousness, and may, indeed, be repressed therefrom.

Psycho-analysts seeking to dissect the unconscious mental forces of the mind have met with a criticism similar to that experienced by those who began the dissection of the human body. It is probably essential to evolution that any attempt intellectually to reverse the process, to reopen (as Samuel Butler would say) questions long since settled into instincts, should seem outrageous, which perhaps explains why many charms and incantations credited to witches took the form of reciting sacred writings *backwards*. The apparent tracking of every affective state to a subconscious sexual source has again led to misunderstanding; but once it is realised that in fact, no less than in the writings of Freud, sex and life are virtually interchangeable terms, the incongruity vanishes.

Again, it is important to be reminded of the neglected truth which Mr. Flügel well states in his sixth chapter, that "the distinction between normal and abnormal is one which is drawn for the sake of practical convenience only, and which indicates merely a difference of degree, not a difference in quality, between the phenomena which it distinguishes." It is probably fair to say that psycho-analysis has made us more frankly aware of this than we were before its method was employed. The study of the family from this point of view is too technical to be summarised in a brief review; the terminology of the analysis itself requires attentive explanation.

Mr. Flügel concludes his book, which shows a wide acquaintance with the literature of the subject, with a statement of its practical value. "When we have brought to consciousness (he remarks) the hidden motives that lurk in the buried strata of our mind, our practical judgment and our reason have a grasp of the psychic situation of such a kind as was before impossible." This, indeed, is the impression left upon the reader by his book. A good deal of the matter is familiar from ordinary observation and reflection, translated, as it were, into the terminology of the new science; and these familiar observations seem to weight those unveilings of the unconscious which the psycho-analytic method has brought forward.—O. B.

**AN INDEX OF TREATMENT.** By various writers. Edited by Robert Hutchison and James Sherren. Eighth edition. (John Wright & Sons, Ltd., Bristol; 42s. net.)

Previous editions of this book are so well known that the present volume requires little introduction. It affords in a convenient form authoritative and up-to-date accounts of the treatment of both medical and surgical conditions. Several articles have been entirely re-written; many new articles have been included, *e.g.*, encephalitis, transfusion, and the surgical treatment of constipation. Special mention may be made of the articles upon the treatment of foreign bodies in the air-passages, insomnia and diabetes. Upon the vexed question of psycho-analysis sound views are expressed without the exaggeration of the fanatic. Prescribing is the subject of a very useful article into which much information has been packed. The subjects are arranged in alphabetical order and there is a reliable supplementary index of cross-references. This is the sort of book which the man who sees it immediately takes steps to obtain and thenceforth keeps within easy reach.—C. E. S.

**OUR BABY: FOR MOTHERS AND NURSES.** By Mrs. J. Langton Hewer. (Simpkin, Marshall; 2s. 6d. net.)

The seventeenth edition of this well-known and extremely useful little handbook on babies is now published, completing a total issue of 160,000 copies since its first appearance twenty years ago. In spite of many competitors for public favour, its popularity has never wavered, and now in its fully revised form, with some parts largely rewritten, it is more than ever a model of what a nursery manual should be, while certain alterations and additions add considerably to its value. It is a book that should be used in every Infant Welfare Centre, and is so arranged that parts of it could readily form the basis of short " talks " to mothers. Such sections as " The Expectant Mother " and " The Importance of Breast Feeding " are good examples of these. The little volume is thoroughly up to date.

**OLDEN TAUNTON.** By Henry J. Alford, M.D. (Woodley & Co., Taunton; 1s. net.)

Dr. Alford, an old resident of Taunton, who has watched for half a century its sanitary development, here records the changes that he has witnessed in his native town. Since his youth—he was born in 1836—he has seen the stage coaches come and go, the hanging in public of a criminal outside the old gaol, and sedan chairs plying in the streets. When appointed medical officer of health, he discovered that nearly all the drinking water used in the town, and derived from wells, was polluted with sewage. He enlarges upon the open and ubiquitous sewers, which preceded the water-carriage system, and polluted the rivers. He recalls the controversy over the best means of sewage disposal, and how the Taunton councillors of the time failed to distinguish between magnesium chloride and sodium chloride as a precipitant with the inevitable consequences. Imperfect or non-existent sanitation led to epidemics. In Dr. Alford's boyhood the only school was " a damp shed." In 1876 he advised that an isolation hospital should be established; and this was opened in 1879. His memories include the old type of nurse. When a student at University College, the head nurse of the ward in his charge was "a tall, gaunt, uncanny, middle-aged woman, with a large aquiline nose, up which she was always shovelling large quantities of snuff." Another of his nurses, at Taunton, was "busy before the fire, warming the ice-bag which he had ordered to be applied to the head of a delirious patient." Though his pamphlet is brief, Dr. Alford's memories throw many interesting sidelights upon the social customs of his time, which are well worth the space which the "Somerset County Gazette" originally devoted to them.—O. B.

**THE PREVENTION OF MALARIA IN THE FEDERATED MALAY STATES.** By Malcolm Watson, M.D., C.M., D.P.H. With a preface by Sir Ronald Ross, K.C.B., &c. Second edition, revised and enlarged. Pp. 381. (John Murray; 36s. net.)

As a record of the triumph of human knowledge over hostile natural forces the story of the great anti-malarial campaign, made possible as the outcome of the truth unearthed by Sir Ronald Ross, is one of the most fascinating in the annals of medical science. Dr. Malcolm Watson, whose untiring efforts in the cause of public health in Malaya are well known, has rendered signal service to the profession in publishing a second edition of his book, the keynote of which lies in the fact that the destruction of anopheles is always effective in reducing or ridding malaria from an infected zone. The operations of drainage and oiling are both described and their effects compared, the former having the advantage over the latter in that it requires less European supervision, for " one can see at a glance in walking over a ravine whether or not it is dry; but one cannot check the work of the oiling coolie so easily." To be a successful administrator in a malarious district the medical officer of health must learn something about drainage of the soil, just as the engineer who is asked to co-operate with him should know something of entomology.

With regard to the use of quinine the author has not seen any material reduction in liability to infection due to its use alone, but when given systematically it " probably assists the infected to acquire a natural immunity." He favours the administration of from ten to twenty grains in solution for three months in cases where there is no reinfection. When personally attacked he preferred a simple watery solution of the bihydrochloride, flavoured with orange or ginger, in a twenty-grain dose divided into three parts and taken at meals.

Writing as an acknowledged optimist, Dr. Watson believes that the solution of the malaria problem, so far as the Federated Malay States are concerned, will also be the solution of the labour problem, and the results of his twenty years' investigations, as recorded in this interesting volume, are sufficient justification of his faith in the development of the country under the British. There are numerous photographs and plans which add greatly to the usefulness of the book.—G. N. M.

